# The Organizer and Its Signaling in Embryonic Development

**DOI:** 10.3390/jdb9040047

**Published:** 2021-11-01

**Authors:** Vijay Kumar, Soochul Park, Unjoo Lee, Jaebong Kim

**Affiliations:** 1 Department of Biochemistry, Institute of Cell Differentiation and Aging, College of Medicine, Hallym University, Chuncheon 24252, Korea; vijay10187@gmail.com; 2 Department of Biological Sciences, Sookmyung Women’s University, Seoul 04310, Korea; scpark@sookmyung.ac.kr; 3 Department of Electrical Engineering, Hallym University, Chuncheon 24252, Korea

**Keywords:** organizer, TGFβ/activin, nodal, BMP signaling, germ layer specification, embryonic development

## Abstract

Germ layer specification and axis formation are crucial events in embryonic development. The Spemann organizer regulates the early developmental processes by multiple regulatory mechanisms. This review focuses on the responsive signaling in organizer formation and how the organizer orchestrates the germ layer specification in vertebrates. Accumulated evidence indicates that the organizer influences embryonic development by dual signaling. Two parallel processes, the migration of the organizer’s cells, followed by the transcriptional activation/deactivation of target genes, and the diffusion of secreting molecules, collectively direct the early development. Finally, we take an in-depth look at active signaling that originates from the organizer and involves germ layer specification and patterning.

## 1. Introduction

In 1924, to understand the processes involved in developmental biology, Spemann and Mangold transplanted a blastopore lip between different ectodermal regions of amphibian embryos [1]. The transplanted dorsal tissue differentiated mostly into a notochord, while the ectoderm of the host dorsal tissue that was sitting above the transplanted region (blastopore lip) was induced and differentiated to form a Siamese twin containing dorsal tissues such as somites and a neural plate, which would form the central nervous system, forming the bulk of a second axis [1,2]. The major findings were that the transplant had altered the fate of the overlying cells and that the neural folds were built from recipient cells and not donor cells. Spemann and Mangold discovered the organizing center in the dorsal blastopore lip of amphibians [1]. This center consists of a cluster of cells in the developing embryo that have the ability to interact and instruct morphogenesis in the surrounding cells during gastrulation [3]. When transplanted to the ventral side of the embryo, the center will induce the formation of a secondary axis, promoting the development of the central nervous system, organs, and tissues, as well as the formation of the main body axis [3,4,5]. Spemann and Mangold found the first evidence of the organizing center, thereafter called the “Spemann organizer”, and its major role in the development of vertebrates. This discovery also introduced the concept of induction in embryonic development, which refers to the method used by specific cells to affect the fate of other embryonic cells [1,2,3]. A major milestone had been achieved for developmental biology.

**Figure 1:**
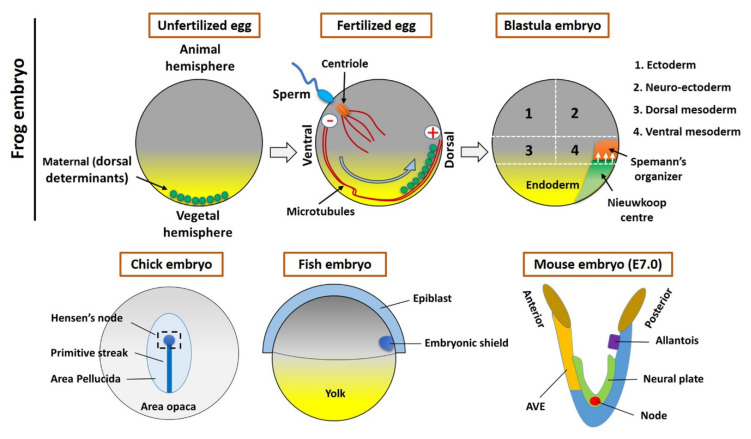
Organizer formation and fate map of *Xenopus* embryos (Upper panel). In an unfertilized egg, the maternal determinants (small vesicles) are located in the vegetal hemisphere. The sperm entry triggers the fertilization process and small vesicles associated with kinesin (motor protein) moved toward the opposite site (the plus end of microtubules) of the sperm entry point. These vesicles contain the components of Wnt signaling, which establishes the Nieuwkoop center and Spemann organizer during the cleavage stages of early embryogenesis. The lower panel shows the Hensen’s node (Chick embryo), embryonic shield (Fish embryo), and node/AVE (Mouse embryo).

Years after the findings of Spemann and Mangold, Harland and Gerhart [6], using the amphibian *Xenopus laevis,* further studied the functions of the organizer and concluded the following. First, the organizer deactivates or activates the surrounding cells to differentiate and start morphogenesis. Second, the three germ layers (endoderm, mesoderm, and ectoderm) of the developing embryo are signaled and affected by the organizer. Third, the three germ layers will respond to the organizer’s signals [6]. Since the initial research carried out mainly by Spemann and Harland, in recent decades many experiments have been carried out to provide more knowledge about the function of the Spemann–Mangold organizer during embryonic development [3,5,7,8,9,10]. It has been found that the amphibious Spemann–Mangold organizer has developmental analogues in other vertebrates [11]. In teleost fish this is an embryonic shield, in avian embryos it called Hensen’s node, and in mammals refer as node. However, the AVE (anterior visceral endoderm); an extra embryonic tissue also known as second organizer in mammals (Figure 1) [12,13,14]. This signifies that the results obtained by studying the Spemann–Mangold organizer can be extrapolated to all vertebrates [5]. All vertebrates are thought to have an organizer that releases inductive signals, responsible for body plan arrangement [5]. The organizer’s distinct cell populations differentiate into various tissues and can be subdivided into head, trunk, and tail organizers based on their different inducing abilities [15]. The literature has found that many molecules are involved in the inductive and organizing properties of the organizer in gastrula cells and consequently in the formation of the three embryonic germ layers [16,17]. Therefore, this review aims to better understand the complex interaction and signaling between the organizer and the embryonic patterning of distinct germ layers, which is a critical step in the development of all vertebrates.

## 2. The Spemann’s Organizer and Homologous Tissue in Vertebrates

In all vertebrates, the organizer (or equivalent) is functionally conserved. The transplanted Hensen’s node can induce neural tissue in fish, mammalian, and amphibian embryos [3,12,18], which indicates that homologous tissue might be functionally conserved among vertebrate species. In this section, we mainly focused on embryonic processes involved in forming amphibian organizer. Additionally, a brief discussion regarding the equivalent tissue in vertebrates is provided.

### 2.1. The Maternal Determinants Establish the Organizer

The formation of Spemann’s organizer involves a series of complex intercellular events that begin right after fertilization. The overall process of organizer formation can be described by some sequential/parallel embryonic events that can be described as two steps. Cortical rotation is the first intracellular event in the fertilized egg that allows for the shifting of maternal determinants from the vegetal to the animal region of the embryo (Figure 1), which establishes the dorsal side (the opposite to sperm entry) [6,10]. The dorsal determinants located in membrane vesicles in the vegetal pole of the embryo are transported to the dorsal side by cortical microtubules and kinesins (Figure 1) [10]. These vesicles are associated with Dishevelled (Dvl/Dsh), a component of the Wnt signal transduction pathway. Therefore, the Wnt pathway will be activated, which will lead to the accumulation and stabilization of β-catenin on the dorsal side (dorsal blastomeres) (reviewed in [10]). This is tightly regulated by the Adenomatous Polyposis Coli (APC), Glycogen synthase kinase 3 (GSK3), and Axin to form a destruction complex and induces phosphorylation-mediated ubiquitination and proteasomal degradation [18,19]. Activation and binding of Wnt ligands to Frizzled (FZD) receptors and co-receptors of the LDL Receptor-Related Protein (LRP) inhibit GSK3 and the destruction complex; hence, β-catenin can accumulate, translocate, and stabilize in the nucleus of the dorsal cells of a blastula stage embryo [20]. β-catenin is required for the activation of initial organizer marker genes like *goosecoid* (*gsc*), *chordin* (*chrd*), *noggin* (*nog*), and *Xnrs* [16,21]. The second event includes the signal generated by maternal factors like β-catenin; these maternal factors activate dorsal determinants. Furthermore, the maternal T-box transcription factor VegT and the maternal TGF-β-family growth factor Vg1 are located on the vegetal hemisphere [22]. At the midblastula stage, mesoderm induction begins, which activates many mesoderm- and endoderm-specific genes in the dorsal and ventral domains of the marginal zone [6]. On the ventral side of the embryo, VegT and Vg1 downregulate the levels of nodal-related genes to originate the ventral mesoderm [3,6,23,24]. Conversely, on the dorsal side, the Wnt/β-Catenin levels coupled with VegT and Veg1 induce the homeobox transcriptional activator Siamois (Sia)/Twin (Xtwn) in the blastula Chrd and Nog-expressing (BCNE) center and regulate the expression of nodal-related genes including *Xnr1*, *Xnr2*, *Xnr4*, *Xnr5*, and *Xnr6* [11,25]. In particular, *Xnr5* and *Xnr6*, acting in combination with Wnt/β-catenin signaling, form the Nieuwkoop center (NC) in the dorsal–vegetal region (Figure 1) of the blastula [26]. Consequently, the NC with Sia and Xtwn will induce the expression of pre-organizer genes such as *gsc*, *Frzb-1*, and *dickkopf-1* (*dkk-1*) [27]. Finally, the Cerberus (*cer*) gene will be upregulated, promoting the formation of overlying mesoderm and the establishment of the Spemann’s organizer, during gastrulation, at the dorsal mesoderm of the embryo [11,25].

### 2.2. The Homologous Structure of Organizer in Other Vertebrates

All vertebrates have a position-specific group of cells (collectively refer as organizers) that are absolutely essential in embryonic development. During embryonic development of the chick, after the mid-streak stage, the top region of the streak thickens and forms the Hensen’s node. Later on, these cells assembled to the midline of the mesoderm to form a notochord [28]. Whereas the embryonic shield forms at the blastoderm margin region (future dorsal side) within the fish embryo, it resembles the dorsal lip of the amphibian embryo [29]. A streak is located on the posterior side of the mouse embryo, wherein the AVE is located at the anterior side. The node located on the ventral surface (during embryonic day 7 (E7)) (Figure 1) [30,31]. This species-specific organizer (s) secrete molecules are most common across the vertebrates, including BMP, Wnt, and Nodal inhibitors [6], and forms prechordal plate and notochord [12,32]. Several studies reported that the organizer’s cells mostly differentiate into axial mesoderm and notochord (or chordamesoderm) [6,8,14,31,33]. However, the organizer influences fate of surrounding cells (or tissues) by extracellular secretion of active molecules [3,6,14]. During development, the organizer (or homologous) tissue transforms into notochord and prechordal mesendoderm (or derivate) that involves several signaling pathways to acquire final fate [6,34,35,36]. However, in the present study, we focused on organizer-induced or mediated embryonic development.

## 3. Overview of Major Signaling Pathways and Targets Involved in Organizer-Induced Embryonic Development

In recent years, a wide range of secreted molecules involved in embryonic development have been isolated and identified from the Spemann organizer using several different screening methods (e.g., cDNA macroarrays) [22]. This has been accomplished mainly in amphibians, but also in fish, birds, and small mammals [37,38,39,40]. The organizer expresses numerous specific genes that encode transcription factors and secreted molecules, involved in complex regulatory pathways of the inducing activity of the organizer [7]. Some of the expressed transcription factors include Gsc (the first molecule expressed in the organizer to be discovered), Sia, dharma, Xtwn, Pintallavis, Xotx2, Xlim1, Xbra, Xanf1/HNF3-β, Lim1, and Xnot, which are homeodomain proteins [2]. Gsc and Sia are exclusively expressed in the organizer, while others such as Xbra and Xnot are initially expressed throughout the entire marginal zone and later become restricted to the organizer [41]. All these components will regulate the expression of the secreted factors, which will then pattern the nearby cells [6]. Other factors include bone morphogenetic proteins (BMPs) antagonists, Chrd, Nog, Follistatin (Fst), ADMP, Xnr-1,-2,-3,-4, Cer, Antivin/Lefty, Frzb1, sFRP2, Crescent, Dkk1, and eFGF, which are able to induce embryonic cell differentiation [7,10]. These molecules act as antagonists for three classes of growth factors (BMPs, Wnts, and Nodals) by interacting directly with the growth factors or with their receptor in the extracellular space [37]. The only exception is Dkk1, which binds the Wnt coreceptor LRP6, instead of growth factors, and, together with another transmembrane protein (kremen), induces endocytosis of the Wnt coreceptor and depletes it from the cell surface [37]. These interactions inhibit the factors, preventing them from signaling [10]. For instance, Chrd and Nog directly bind and prevent BMPs from binding to their cognate receptors, promoting anterior development [42]. Dkk1, Frzb1, and Crescent are antagonists of Wnt/β-catenin, the inhibition of zygotic Wnt signaling blocks ventralizing and posteriorizing activity and promotes dorsoanterior development and pattern the neural plate. While, Antivin/Lefty binds to the TGFβ/Nodal receptor and inhibits Nodal signaling [11,20,43,44]. This means that the Spemann organizer is a negative regulator of some signaling, including Wnt, BMP, and Nodals [45]. However, the expression of all these negative regulated molecules by the organizer generates a signaling gradient, together with the ventral signaling center, that is responsible for shaping the dorsal–ventral and anterior–posterior patterning of the embryonic axes [46]. In this section, we have briefly described the major signaling and molecular mechanisms involved in the activation/deactivation of target genes.

### 3.1. Wnt/β-Catenin Signaling

**Figure 2:**
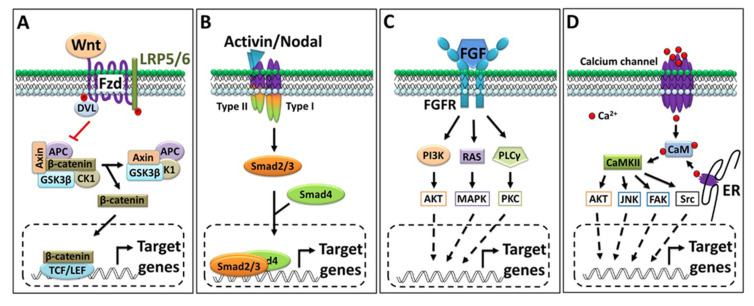
Outline of key signaling pathways. (**A**) Canonical Wnt signaling pathway; on a signal (Wnt) input, Dvl inactivate the destruction complex and releases β-catenin. β-catenin translocates into the nucleus, where it can interact with a transcription factor to regulate the target gene’s expression. (**B**) Activin/Nodal signaling pathway. Activin and Nodal bind to the same set of receptors that activate the intracellular domain of the receptor and sequentially activate Smad2/3. (**C**) FGF signaling pathway: depending on the ligand, FGFR activates different types of cytosolic effector proteins. (**D**) Ca^2+^ signaling pathway: intracellular calcium interacts with a calcium binding protein that activates intracellular protein kinase and ultimately regulates the target gene’s expression via several sets of transcription factors.

The functional role of the Wnt signaling pathway is highly conserved across vertebrates and plays a central role in developmental biology [47]. Wnt signaling is divided into canonical (β-catenin-dependent) and noncanonical (β-catenin-independent) types. The noncanonical Wnt signaling has two branches known as the Wnt/PCP and Wnt/Ca^2+^ pathways [19]. In the canonical pathway, the interaction of Wnt (ligand) with the membrane-bound receptor Fzd (frizzled) and LRP5/6 (lipoprotein receptor related protein 5/6) stimulates the cytosolic protein Dishevelled (Dsh or Dvl) to interact with Fzd. In unstimulated conditions (an inactive Wnt receptor), the destruction complex (a multiprotein complex; generally, Axin, APC, CK1, and GSK3β) remains active and phosphonates the β-catenin (by GSK3β), which triggers E3 ligase-mediated proteasomal degradation [47,48]. Wnt interacts with and activates the receptor and cytosolic Dsh, which ultimately inactivates the destruction complex. Once the destruction complex is inactivated, β-catenin is released and translocates into the nucleus to regulate the target gene’s expression (Figure 2A) [47]. The nuclear β-catenin physically interacts with several transcription factors like Tcfs and promotes dorsoanterior fate specification [48,49]. In the noncanonical Wnt/PCP pathway, Dsh activates a different set of intracellular effector proteins (such as Daam1 and Rac1); these effectors then modulate the other sets of kinase(s) and transcription factors to activate/deactivate a different set of genes [19,50]. In the Wnt/Ca^2+^ pathways, Fzd transduces signals to phospholipase C, which leads to the activation of several intermediate components of Ca^2+^ signaling [19]. Since β-catenin is a maternal factor located in the dorsal region of embryos, it plays a master role in organizer establishment. β-catenin induces the expression of most organizer genes in vertebrates [48]. Some studies have demonstrated that maternal Wnt/β-catenin targets upstream signature organizer markers including *gsc*, *siamois*, and *twin* in the *Xenopus* embryo [51]. However, the identification of a genome-wide target of maternal Wnt/β-catenin showed a broad range of target genes that include transcription factors, receptors, and inhibitors like *tcf7*, *hes-like*, *kremen2*, *vegt*, *fzd10*, etc. [52].

### 3.2. Activin/Inhibin and Nodal Signaling

Transforming growth factor β (TGFβ) comprises 33 members and includes the activin, inhibin, and nodal families of ligands. Typically, the three subunits (α, βA, and βB) dimerize (homo- or heterodimer) in different combinations to produce a functional dimer. Inhibin consists of a common α-subunit and always a second βA-subunit or βB-subunit to dimerize. Therefore, the only two possible dimer combinations are inhibin A (α-subunit and βA-subunit) and inhibin B (α-subunit and βB-subunit) (reviewed in [53]). Summarily, the functional activin ligand is a dimer of two identical or distinct monomers that dimerize to form a homo/heterodimer, activin A (βA βA) and activin B (βBβB)m whereas the heterodimer forms as activin AB (βAβB) (reviewed in [53]). The nodal ones are another type of ligands belong to the TGFβ family and actively involved in mesendoderm patterning during embryogenesis via the same membrane-bound receptors [54]. To date, several nodal (or related) ligands have been characterized in vertebrate models (reviewed in [54,55]). These ligands generally interact with a set of type I activin receptors (ALK1-7) and type II activin receptors (ActRII/IIB). The type II (ActRII/IIB) receptors are common in the receptor complex for activin, inhibin, and nodal. However, type I (ALK1-7) in the receptors complex recognized by activin A, activin B, and activin AB, for inhibin type II (ActRII/IIB), generally forms a complex with a co-receptor (β-glycan), and the nodal co-receptor (EGF-CFC) frequently interacts with type I (ALK1-7) [53,54,55,56,57]. The ligands binding to these receptors ultimately activate the intracellular domain (serine/threonine kinase) of receptors; in turn, the activated receptor then phosphorylates the (activational) Smads (typically, Smad2/3) family of transcription factors to initiate developmental processes [56,57]. A recent study showed that Smad2/3 activates *chrd* transcription by directly binding to ARE (activin response elements) within the promoter region [58]. However, activin induces most organizers and other mesendoderm and neural genes’ expression is widely accepted now [59,60,61,62].

### 3.3. FGF Signaling

FGF signaling plays an essential and diverse role in the overall embryonic development of vertebrates. A recent study found that the total number of reported FGF ligands in mice and humans is 22, wherein *Xenopus* 19 ligands have been identified [63]. Furthermore, these ligands prefer one (or more) FGF receptors (FGFR1–4) to interact with, and FGF/FGFR interaction eventually activates the cytosolic effector protein(s) shown in Figure 2C. As to the organizer, the expression of several types of FGF/FGFR is reported in the Spemann organizer or mesodermal region of *Xenopus* embryos. The expression of *fgf4*, *fgf8*, and *fgf20* was reported at a high level in the mesodermal region of *Xenopus* embryos. In addition, several mesodermal markers including *xcad2* and *xbra* showed marked responses to FGF signaling; we refer readers to these particular articles [63,64,65,66].

### 3.4. Calcium Signaling

The intracellular Ca^2+^ concentration is directly modulated by influx (from the extracellular space or endoplasmic reticulum), mediated via several types of Ca^2+^ channels. The intracellular Ca^2+^ interacts with and activate a variety of calcium-binding effector proteins (e.g., Calmodulin (CaM)) and Ca^2+^-dependent protein kinase (CDPK) [67,68,69]. Collectively, CaM and CDPK activate several sets of other CaM kinases (CaMKs); once CaMKs are activated, they can modulate several other intermediate signaling molecules (Figure 2D) and transcription factors to regulate cell fate and differentiation [67,68,69,70]. In recent years, many important reports have demonstrated that Ca^2+^ is essential for involuting/migrating mesodermal cells [71,72]. However, several reports suggest that Ca^2+^ signaling interacts with other pathways and plays an inductive role in neural development in vertebrates; for details, readers may wish to consult the cited articles [71,73,74,75,76].

## 4. The Organizer as an Organizing Center of Vertebrate Embryonic Patterning

**Figure 3:**
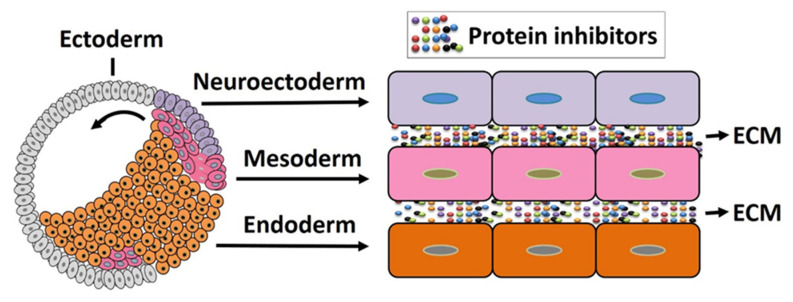
Early gastrulation in amphibian embryos. The dorsal mesoderm (organizer) cells initiate migration from the dorsal blastopore lip and establish primary germ layers. The involuting cells release several protein inhibitors that modify the surrounding cells’ fate.

The Spemann organizer is a dynamic and heterogeneous structure composed of distinct cell populations that make it a suitable candidate to serve as a master regulator of embryonic development in vertebrates. The active protein molecules secreted from the organizer are mostly inhibitors of BMP, nodal, Wnt, and other signaling that enable self-differentiation as well as modulate the fate of surrounding cells. During gastrulation, the organizer (dorsal mesoderm from dorsal blastopore lip) cells initiate migration toward the ectoderm layer (animal hemisphere) to form primary germ layers. These migrating cells contact neighboring cells directly or through the extracellular matrix (ECM) (Figure 3). The ECM contains certain types of protein inhibitors that interfere with adjacent cell signaling to drive overall embryonic patterning. In this section, we have briefly discussed the germ layer patterning and embryonic axis formation, with a view to understanding the organizer’s involvement in this process.

### 4.1. Organizer in Mesoderm Formation and Patterning

Once the organizer has been fully formed in the embryo, the tissues that surround the organizer will receive organizer-produced signals that induce transcription factors (e.g., Gsc and Xbra) and morphogen molecules (e.g., FGFs and RA) and create a signaling gradient of BMPs, Wnts, and nodals. Nodal, BMPs, and Wnt signals crossregulate each other, as nodals induce other nodals, Wnts, and BMPs, as well their antagonists [11]. These factors and signals will induce the patterning and formation of germ layers along the dorsal and anterior axis. The mesoderm is the first to be affected by the signals, followed by the ectoderm and finally the endoderm [77]. These inductions generally occur during gastrulation, a process that transforms the organization of the embryonic layers, by means of organized cell movements and rearrangements, arranging the mesoderm between the external layer (the ectoderm) and the internal layer (the endoderm) (Figure 3) [6,12]. Each of these layers contains cells that respond to the organizer’s signals, originating a variety of possible developmental responses. The mesoderm will originate the notochord, axial skeleton, connective tissue, kidneys, blood, somites, trunk muscles, and cartilage [78]. Collectively, the three germ layers give rise to all somatic tissues and organs in vertebrates. The mesoderm is patterned along the dorsoventral axis to give rise to axial (notochord and prechordal mesoderm), paraxial (somites), intermediate (pronephros), and lateral (blood) derivatives [24]. This patterning occurs via an interplay between ventralizing and dorsalizing signals, molecules, and transcription factors secreted by the organizer [15]. A BMP signaling gradient is created throughout the embryo. BMPs are thoroughly expressed in the blastula; however, the organizer antagonizes the levels of BMPs in specific regions, by producing BMP antagonists [15]. Some of these molecules are Nog, Chrd, and Follistatin, which sequester and bind to BMPs from the extracellular space, preventing them from binding to their cognate receptors, thus blocking their activity [42]. This will create a BMP gradient, as seen in studies about the dorsal ventral patterning of the mesoderm. The functional mutations in BMP2 and BMP7 result in strong dorsalization, while chrd mutants are ventralized, suggesting that the BMP gradient results from the interaction of autoregulatory BMPs with dorsal antagonists (e.g., chrd) [79,80,81], which is necessary for mesodermal patterning. In mice, BMP4 mutants fail to develop a mesoderm [82], suggesting the requirement of active BMP signaling for normal mesoderm formation. Since a gradient is created, not all components of mesoderm require a similar concentration of BMP. Indeed, for example, no BMP is necessary for a notochord to be formed; low levels of BMP are needed for muscles and somites, while higher levels are required for the kidneys, lateral plate, and blood [46]. In addition to BMP inhibition, the interaction of Wnt morphogens and Wnt antagonists (e.g., *dkk*-1, *Frzb*-1, and Crescent), expressed by the organizer, generates a Wnt signaling gradient, also required for mesoderm patterning—especially for notochord formation, but not necessary for trunk muscles [15]. In this sense, the double inhibition of Wnt and BMP is able to induce a notochord [83]. Lastly, the Nodal signaling gradient, through antagonists (antivin/lefty), is also essential for inducing mesodermal tissues [78]. Amphibian embryos express many nodal relatives (Xnr1, -2, -4, -5, -6), which can heterodimerize and induce mesoderm [78]. In an experiment, when the antinodal reagent Cer is injected, nodal signaling, antivin (a nodal antagonist), and consequently mesoderm formation are inhibited [54]. The low levels of activin, using morpholino antisense nucleotides, affected mesoderm formation in the frog embryo [84]. In mice, using mutants, nodal signaling experiments provided the same results as in fish and amphibians, confirming that the nodal pathway is essential for mesoderm formation [85,86]. In zebrafish, the overexpression of high doses of antivin deplete the axial, paraxial, and ventral mesoderm [87]. Other studies showed that the nodal signaling gradient regulates patterning along the anterior ventral axis [54]. High levels of nodal signaling induce the prechordal marker *gsc*, whereas lower levels induce the notochord marker expression [23]. Similarly, the reduction in nodal signaling by expressing the nodal antagonist lefty converts the mesoderm into notochord [23,88]. These results indicate that nodal signaling patterns cell fates along the anterior ventral and dorsal ventral axis. Interestingly, a nodal gradient is essential to set up the other two secondary signaling gradients of BMPs and Wnts. This has been seen because low nodal signaling induces growth factors that inhibit the organizer (e.g., BMP4 in zebrafish and Wnt8 in *Xenopus*), while higher nodal doses induce the antagonists Chrd, Dkk1, and Cer [27,33,89]. Similarly, activin induces all organizers or mesodermal markers including the *gsc* and *chrd* genes’ expression, in a Smad2/3-dependent manner [58,59]. 

FGFs have also been demonstrated to induce mesoderm formation. In experiments using mouse, fish, frog, and chick embryos, when FGFs’ function was blocked, the mesoderm development was affected [90,91,92,93]. This is because FGFs are required for the cellular response of the transforming growth factor βs (TGFβs) [94,95]. However, residual FGF signaling induces the expression of several key mesoderm markers including *xbra* and *xcad2/3* [63,96,97,98]. Supporting evidence has been collected: when FGF signaling was blocked with dominant negative FGF receptor (DNFR, the embryos failed to produce mesoderm [99]. The FGF signaling interplays with several signaling pathways that have integrated roles in mesoderm formation [63]. Besides the nodal, Wnt, and BMP gradients, the Spemann organizer also expresses various transcription factors (e.g., *gsc*, Pintallavis, Xbra, and Xlim1) involved in mesoderm formation [6]. For instance, Gsc represses Xbra and *Wnt8* expression [100,101]; it has been suggested that this is how different activin doses are translated into different gene expression domains in the mesoderm. Pintallavis synergizes with Xbra to induce notochord and pattern the mesoderm. In the same manner, Xlim1 interacts with Xbra to generate an anterior ventral axis, vital in mesoderm formation [102,103]. Moreover, Xbra interacts with Smad1 to control ventral fate via increasing the expression level of *ventx1.1* and *ventx1.2* [104,105]. Many experiments using fish, frog, and mice embryos have been carried out and revealed that most of the organizer-expressed transcription factors are required, at least to some extent, for mesoderm formation [106,107,108,109].

### 4.2. The Organizer in Ectoderm Patterning

**Figure 4:**
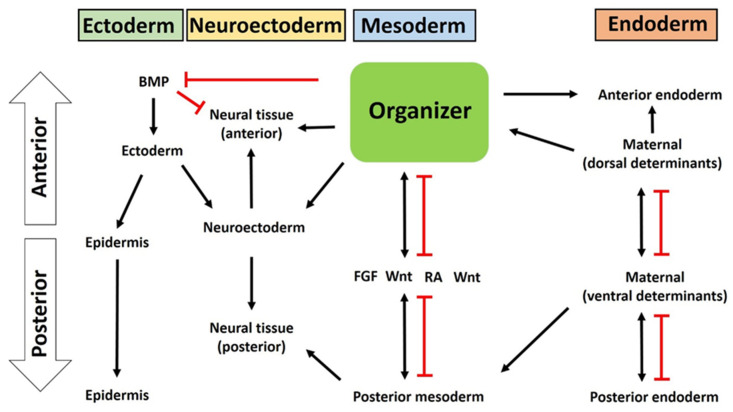
A putative model of how the organizer induces germ layer specification and patterning.

In the embryo, in the early to middle gastrula stage, besides the dorsal signaling center (Spemann organizer), there is also a ventral signaling center [44]. In the ventral region, many genes encoding secreted or cell surface proteins are expressed, including BMP2, 4, and 7, Wnt8, CV2 (Crossveinless-2), Sizzled, Tsg (Twisted Gastrulation), Xlr (Xolloid-Related), and Bambi (BMP and activin membrane-bound inhibitor) [11]. The ventral center has a high level of signaling by BMP4 and BMP7, which bind to membrane BMP receptors (BMPR), which subsequently phosphorylate the C-terminal of Smad1 (transcription factor), activate Smad1, which is translocated to the nucleus, and activate certain target genes critical to early development [105,110]. Surprisingly, the dorsal and ventral centers antagonize each other. Several components that are expressed in the ventral center, and that are transcribed when BMP signals are high, have counterparts in the dorsal center, in which they are transcribed when BMP signals are low [2]. Furthermore, dorsally secreted antagonists and transcriptional repressors prevent the activity and expression of ventral morphogens, protecting the dorsal side of the embryo from their ventralizing and posteriorizing activities [5,12,58,78,111,112]. For instance, dharma represses BMP-2, while Gsc decreases the transcription of Wnt8. Similarly, the molecules expressed in the ventral side prevent the expression of dorsally secreted molecules in that territory [106,111]. For example, CV2 is an agonist of BMP signaling when bound to Chrd or the Chrd–BMP complex [42]. Moreover, Tsg binds to BMP and Chrd, forming a ternary complex when high levels of xolloid (a secreted metalloproteinase) are present, in which Tsg enhances BMP signaling by promoting the proteolytic cleavage of Chrd by Xolloid [113]. Therefore, contrary to the dorsal region, in the ventral gastrula center, the activity of Chrd, the main BMP antagonist, is negatively regulated [5]. Although BMPs are expressed throughout the embryo during the gastrula stages, the interactions between dorsal and ventral signals are vital to create a BMP signaling gradient. In the ventral marginal zone there is a higher level of BMP signaling, while the dorsal side is the region with the lowest BMP signaling [11]. For instance, in *Xenopus* no BMP is required for notochord, low levels of BMP signaling are necessary for muscle, and even higher BMP signaling is needed for the formation of blood and the lateral plate [12,44,46,105,106,114,115]. This is necessary to regulate dorsal–ventral and anterior–posterior patterning in the gastrula, to ensure optimal embryonic development (Figure 4).

### 4.3. The Organizer Induces Neural Induction and Neuroectoderm Formation

During gastrulation, the Spemann organizer is deeply involved in the patterning and formation of ectoderm and neuroectoderm. Years of research have led to the identification of a large number of genes, molecules, and pathways responsible for the inductive properties of the organizer in neuralizing the ectoderm and patterning the neuroectoderm, critical steps for the formation of the epidermis and central nervous system. During gastrulation in vertebrates, cells of the ectoderm can be induced into distinct fates: cells can become flattened and originate the epidermal progenitors (skin epidermis) on the ventral side or turn elongated and give rise to neural progenitors (neural crest) on the dorsal side [76,116]. This change in cell shape causes the prospective neural region to rise and form a neural plate, which will then thicken and form neural folds, move towards the midline of the embryo, and fuse. Beneath the epidermis, the neural tube is formed and the dorsal-most portion of the neural tube will give rise to the neural crest [5]. This differentiation is mediated via the action of inducers, mainly released by the organizer, that result in distinct levels of signaling depending on the fate of ectodermal cells [117]. Now it is widely accepted that the organizer releases potent BMP inhibitors (e.g., Chrd and Nog) whose action promotes neural induction and the phenomenon known as default model neurogenesis [118]. Moreover, several experiments have demonstrated the importance of BMPs in inhibiting neural induction and enhancing epidermis formation. In ectodermal cells, BMP4 suppresses the expression of neural markers and induces epidermal keratin expression [119]. Similarly, BMP2 and BMP7 have also been reported to be neural inhibitors but epidermal inducers [120]. BMP receptors, ligands, and antisense have also produced similar effects in vertebrates [120,121,122]. All these studies revealed the importance of BMP in epidermal specification and neural induction; no BMP is required. Neural induction is a process initiated during gastrulation, by which the embryonic ectoderm surrounding the organizer (or migration mesoderm) gives rise to the neuroectoderm to form the neural plate and nervous system, together with the anterior–posterior pattern and dorsoventral organization, via the direct action of intercellular communication between the Spemann organizer and the adjacent dorsal ectoderm [6]. Recent reports indicates that the organizer plays a dual role in neural induction.

In the first step, the organizer inhibits BMP by releasing several BMP inhibitors. For the formation of the nervous tissue, the levels of BMP need to be significantly reduced in the dorsal part of the ectoderm, which is regulated by BMP antagonists (e.g., chrd, nog, fst, cer, and Xnr3) [76]. These molecules are vital to prevent the binding of BMPs to their receptor complexes, leading to a blocking of BMP signal transduction. Specifically, follistatin interferes with BMPs by the formation of an inactive trimeric complex [123]. This inhibition of BMP signaling results in the activation of neural gene expression [78]. Studies have been performed using several vertebrates to demonstrate the importance of the antagonists in the ectoderm and neuro-ectoderm. In zebrafish, double mutants for the BMP antagonists, *chordino* (a Chrd homolog) and *ogon* (a secreted Frizzled homolog), do not develop a nervous system [124,125]. Mice and *Xenopus tropicalis* mutant embryos that lack chrd, nog follistatin, and cer fail to develop an anterior brain and neural structures [107,126]. Cer-dependent neural induction has been shown to be inhibited by BMP4 [127]. Moreover, BMP4 also blocks xnr3-induced neural formation [128]. Growth and differentiation factor-6 (GDF6), from the TGF-β family, induces epidermis development and blocks the formation of the nervous system [76]. GDF6 can create complexes with BMPs to regulate embryonic development. However, it seems that nog can interact directly with GDF6, blocking its activity [129]. BMP-targeted genes (e.g., Msx1, Gata2, Vent1, and Vent2) are important for epidermal tissue development in the ectoderm. Injection of *Msx1* mRNA inhibited the neural tissue formation induced by nog [130]. Overexpression of Vent1 or Vent2 induces epidermis development and inhibits the formation of neural tissue [131]. Additionally, both Vent proteins are required to restrict the expression of geminin, an early neural transcription factor, to the ventral ectoderm [111,131,132]. Geminin’s main function is to regulate the levels of BMP during the gastrula stage at the dorsal ectoderm, by acting on neural genes (e.g., Xngr1), ensuring that BMP levels are minimal to allow the formation of the neural plate. Moreover, if geminin is incorrectly expressed in the dorsal ectoderm, it produces islets of epidermal genes’ expression within the neurectoderm [132]. Therefore, geminin plays a vital role in early development to decrease the expression of BMPs and, consequently, impact the development of neural tissue [76]. The inhibition of BMP signaling is enough to induce neural tissue in the ectodermal explants; however, it is not sufficient to induce it in the non-neural ectoderm of the embryo [121,133]. This has been seen when BMP signaling was inhibited in the ventral ectoderm using Smad6 (which repressed epidermis formation) but failed to induce the expression of neural markers [134,135]. Furthermore, whereas BMP is necessary and sufficient to inhibit neural fates in mammalian embryos, it is not sufficient in chicken embryos [114,136,137]. Therefore, it has been speculated that other signals and pathways are involved in these processes but they also need to be silenced by the organizer to allow for proper development of the embryo [138]. 

In the second step, at the same time the organizer also produces inductive molecules that induce neural-specific genes’ expression, parallel to BMP inhibition. BMP inhibition itself activates some neural specifiers, for example Foxd411.1. Once Foxd411.1 is produced, it drives further neural development [139]. Some inductive signals work together to induce neural induction and promote neural fate acquisition (Figure 4). Noticeably, Zic3, Zicr1, and XlPou2, expressed in the neurectoderm at the gastrula stages, also participate in neural induction and are induced by BMP antagonists (Nog and Chrd) [117]. Similarly, Nog induces the interaction between Trpc1 (transient receptor potential canonical subfamily member 1, a calcium channel) and BMPRII (BMP receptor type II); this complex allows for the activation of several Ca^2+^ channels and cations influx. Increased amounts of intracellular Ca^2+^ activate Ca^2+^-mediated signaling pathways and activate neural genes’ expression [73,76]. However, Wnt antagonists are also important to maintain low Wnt activity in the anterior region, inhibit BMP transcription, and establish an endogenous Wnt/β-catenin signaling morphogen gradient along the neural axis, critical for neural development [11]. For instance, Dkk1, combined with a BMP inhibitor, induces a prechordal plate [140]. Also, when Dkk1 was used to inhibit zygotic Wnt signaling, it induced ectoderm explants to express anterior neural markers [141]. Furthermore, in chick embryos, overexpression of Wnt3a inhibited the neuralization of ectoderm by BMP inhibition and FGF signaling, while promoting epidermal fates [142]. Moreover, many Wnt/β-catenin target genes have been identified, such as Xcad3 and Meis3, relevant for ectoderm and neuro-ectoderm induction [143,144]. Members of the FGF family are morphogens for neural induction. FGFs activate a mitogen-activated protein kinase (MAPK) cascade that results in the phosphorylation and inactivation of a crucial linker region of the BMP effector Smad1, which represses the transcription and pathway of BMPs, promoting epidermis formation, as seen in zebrafish, chicks, and frogs [138,145,146,147]. Therefore, FGFs works in cooperation with BMP antagonists. In experiments where FGF was inhibited, neural induction and loss of the anterior neural tissue failed to produce ectodermal explants [135,148]. In support, several studies indicated that the same FGF (for example, FGF4 and FGF8) may act as an instructive molecule to activate neurogenesis [75,137,139,149]. Similarly to Wnt signaling, the functions of FGF are mediated by the transcriptional activation of a cascade of posterior genes, such as Xcad3, Hoxb9, and Hoxa1, in the early neurula stage [150,151]. FGF also functions in cooperation with Wnt signaling to pattern the neural axis through the regulation of posterior gene expression [152]. Taken together, these results indicate that the organizer has a dual nature (inhibitory for BMP and instructive for neural fate) in neuroectoderm formation (Figure 4).

### 4.4. The Organizer Is Involved in Endoderm Patterning

The endoderm is the innermost germ layer. Similar to in the ectoderm and mesoderm, as a distinct signaling center, the organizer affects endoderm patterning by a gradient of extracellular molecules. During the blastula stage, the NC contains maternal determinants that actively influence the organizer’s formation and at the same time determine endodermal cells’ responses to these signals. Once the organizer cells start migrating, the overlapped endodermal cells internalize with the mesoderm (Figure 3), which then leads to the formation of the dorsoanterior endoderm. In the neurula stage of *Xenopus* embryos, the majority of the endodermal mass elongates along the anterior posterior endodermal axis and forms a primitive gut tube [153]. During this elongation process, the Wnt/PCP and Wnt/β-catenin pathways remain active and mesoderm is inhibited by Sfrp5 by (Wnt antagonist) in the endoderm, while Wnt inhibition induces foregut morphogenesis [154]. In the mouse embryo, anterior endoderm emerges from a primitive streak (an organizer homologue) that differentiates into the foregut [153]. However, the anterior foregut, midgut, and posterior hindgut receive a different gradient of signals from the mesoderm that collectively drive endoderm patterning. Indeed, for example, the anterior-most (foregut) received high activin signals but lower Wnt, FGF, and BMP. On the contrary, the posterior (hindgut) received a high amount of Wnt, FGF, and BMP but less activin; for details, readers are referred to these targeted articles [153,154,155].

## 5. Conclusions

We have discussed the possible roles of the organizer in the early embryonic development of vertebrates. In the present review, we have focused on embryonic patterning and how several signaling pathways engage in crosstalk to drive proper embryonic patterning in a spatiotemporal manner. The organizer itself regulates the expression of several sets of genes that encode secreted growth factors and antagonists. Collectively, these growth factors/antagonists activate or deactivate the target signaling in the surrounding germ layer in coordination with normal embryonic development. Finally, we collected the integrated signaling cascade that originated from (or was involved with) the organizer and plays an important role in establishing the overall embryonic axes.

## Data Availability

Not applicable.
